# Spatial distribution of cutaneous leishmaniasis in the state of Paraná, Brazil

**DOI:** 10.1371/journal.pone.0185401

**Published:** 2017-09-22

**Authors:** Helen Aline Melo, Diogo Francisco Rossoni, Ueslei Teodoro

**Affiliations:** 1 Postgraduate Program in Health Sciences, Universidade Estadual de Maringá, Maringá, PR, Brazil; 2 Department of Statistics, Universidade Estadual de Maringá, Maringá, Paraná, Brazil; University of Minnesota, UNITED STATES

## Abstract

The geographic distribution of cutaneous leishmaniasis (CL) makes it a disease of major clinical importance in Brazil, where it is endemic in the state of Paraná. The objective of this study was to analyze the spatial distribution of CL in Paraná between 2001 and 2015, based on data from the Sistema de Informação de Agravos de Notificação (Information System for Notifiable Diseases) regarding autochthonous CL cases. Spatial autocorrelation was performed using Moran’s Global Index and the Local Indicator of Spatial Association (LISA). The construction of maps was based on categories of association (high-high, low-low, high-low, and low-high). A total of 4,557 autochthonous cases of CL were registered in the state of Paraná, with an annual average of 303.8 (± 135.2) and a detection coefficient of 2.91. No correlation was found between global indices and their respective significance in 2001 (I = -0.456, *p* = 0.676), but evidence of spatial autocorrelation was found in other years (*p*< 0.05). In the construction and analysis of the cluster maps, areas with a high-high positive association were found in the Ivaí-Pirapó, Tibagi, Cinzas-Laranjinha, and Ribeira areas. The state of Paraná should keep a constant surveillance over CL due to the prominent presence of socioeconomic and environmental factors such as the favorable circumstances for the vectors present in peri-urban and agriculture áreas.

## Introduction

Leishmaniasis is a globally distributed disease. Approximately 350 million people are currently at risk of contracting at least one of its variants [1]. Brazil had an annual average of 26,965 registered cases of cutaneous leishmaniasis (CL) from 1993 to 2012, with an average detection coefficient of 15.7 cases for every 100,000 inhabitants [2]. Throughout this period, an increasing trend was observed, with higher coefficients in 1994 and 1995, 22.83 and 22.94 cases for every 100,000 inhabitants, respectively [2]. When analyzing the evolution of CL in Brazil, one noticeable factor is its geographical expansion. At the beginning of the 1980s, autochthonous cases were registered in 19 states. By 2003, every state in the country had registered cases of CL [2]. Since the early 1900s, human cases of CL have been registered in northern, western, and southeastern regions of the state of Paraná. In the northern region of Paraná, the disease reached epidemic proportions between the 1930s and 1950s when the area was experiencing significant immigration [3]. The incidence dropped drastically during the 1950s as a direct result of public campaigns for the eradication of malaria and the use of insecticides [3]. However, since the 1980s, the incidence of CL has returned to endemic proportions in the state of Paraná [2,4].

In Brazil, *Leishmania (Viannia) braziliensis*, *L*. *(Leishmania) amazonensis*, and *L*. *(V*.*)guyanensis* have been the most frequent causes of CL in humans [2]. In Paraná, CL is directly linked to wild transmission cycles of the parasite in natural foci that persist in forest preserve areas and traditional agricultural production zones [5–7]. Cutaneous leishmaniasis persists in the state despite the replacement of natural vegetation with corn, cotton, and pasture plantings, affecting individuals of all age groups and both genders [5–7]. Anthropogenic actions that affected the environment and increased urbanization and socioeconomic pressure may have contributed to an increase in endemic areas and outbreaks in urban areas [5]. In areas that have been modified by human activity, CL has been found in environmental preservation areas with small patches of forest, such as the cities of Maringá [5,8] and Cianorte [5].

Understanding spatial patterns with the use of human risk geoprocessing techniques is important for the proper guidance of prevention, surveillance, and control measures [9–11], based on the assumption that spatially related data samples within close proximity to each other possess similar behavior. The use of geoprocessing techniques and statistical spatial analysis enables the creation of maps that detail the risk of occurrence of CL. Based on these analyses, associations between cases of CL and different degrees of anthropogenic activity can be determined in areas where there is notification of the disease, with the goal of identifying possible patterns between such areas [10]. The aim of the present study was to use statistical spatial analysis in the state of Paraná to evaluate the dynamics of CL occurrence from 2001 to 2015 in an attempt to support planning control measures that can effectively mitigate the impact of the disease on the population.

## Materials and methods

### Study area

The state of Paraná is in southern Brazil (22°30’58” and 28°43’00” S; 48°05’37” and 54°37’08” W). It has an area of 199,307.945 km^2^ and an estimated population of 11,163,018, with a demographic density of 52.40 inhabitants per square kilometer in 2015 [12–13]. Paraná has 399 municipalities that are distributed into 10 macro regions (i.e., geopolitical subdivisions that encompass several municipalities with economic and social similarities) and 39 micro regions (i.e., a group of neighboring municipalities) [12–13].

Paraná has three distinct climatic groups, according to the Köppen climate classification system: (1) Humid Subtropical Climate–Mesothermal (Cfa), with an average high temperature of 22°C that can reach 40°C in the north, west, and Ribeira river valley and an average low temperature of 18°C (this is the most widespread type of climate in the state, (2) Temperature Oceanic Climate–Mesothermal (Cfb), with an average temperature of 18–22°C, and (3) Tropical Rainforest Climate–Megathermal (Af), which is restricted to the coastal strip and has an average temperature above 18°C [14].

### Data collection

To analyze the spatial distribution of autochthonous cases of CL in the state of Paraná, we used data from the Sistema de Informação de Agravos de Notificação (SINAN; Information System for Notifiable Diseases) from January 2001 to December 2015. To calculate the detection coefficient (autochthonous cases per 100,000 inhabitants), we used the estimated annual population and the territorial area of each municipality, based on the Instituto Brasileiro de Geografia e Estatística (Brazilian Institute for Geography and Statistics) [12–13]. We gathered information on gender, age, clinical form of CL, and proportion of CL patients that achieved clinical cure. In the present study, we focused on municipalities with detection coefficients >10.0 because the highest risk for CL transmission in these areas.

### Statistical analysis

The spatial analysis was conducted in three stages. In the first stage, a test was performed to detect spatial autocorrelation and verify global spatial dependency against the incidence of autochthonous cases of CL [15]. In the second stage, the Local Indicator of Spatial Association (LISA) was employed to analyze local spatial association, which produces a specific value for each municipality and allows the identification of clusters of municipalities with local similarities in terms of the incidence of CL [15]. In the third stage, maps were constructed by category, with two possible classes of direct association (high-high and low-low) and two possible classes of negative association (high-low and low-high) [15].

To detect spatial autocorrelation, Moran’s Global Index (Moran’s I) was used, defined as:

$$I_{i}=\frac{\left(y_{i}-\bar{y}\right)\sum_{j=1}^{n}w_{ij}\left(y_{j}-\bar{y}\right)}{\frac{\sum_{i=1}^{n}{\left(y_{i}-\bar{y}\right)}^{2}}{n}}$$

where *y*_*i*_,*y*_*j*_ are the samples collected at points *i* and *j*, respectively, $\bar{y}$ is the mean value, *w*_*ij*_ is the spatial neighboring component, and *n* is the size of the sample.

For LISA, Moran’s Local Index was used, defined as:

$$I_{i}(d)=\frac{\left(x_{i}-\bar{x}\right)}{s^{2}}\sum w_{ij}(d)(x_{i}-\bar{x})$$

where *x*_*i*_ is the sample collected at point *i*, $\bar{x}$ is the mean value, *w*_*ij*_ is the spatial neighboring component, and *s*^2^ is the variance.

All of the statistical analyses were performed with software R environment confidence interval of 95% [16] and with package “spdep” [17–18]. This package provides a collection of functions to create spatial weights matrix objects from polygon contiguities, from point patterns by distance and tessellations, for summarizing these objects, and for permitting their use in spatial data analysis, including regional aggregation by minimum spanning tree.

## Results

**Fig 1:**
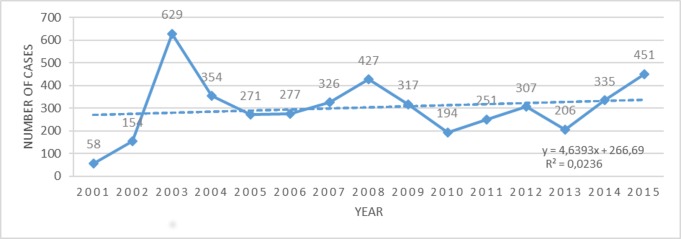
Distribution of cutaneous leishmaniasis cases in the state of Paraná, Brazil, from 2001 to 2015.

From 2001 to 2015, 4,557 cases of CL were diagnosed in the state of Paraná, with an average annual case rate of 303.8 (±135.2), a detection coefficient of 2.91, and a density of 0.023 cases per km^2^. The year with the highest number of cases was 2003 (609 cases) with a detection coefficient of 6.35 (Fig 1). Males between 20 and 59 years of age were most affected, with a predominance of the cutaneous clinical form and evolution of the majority of the treated cases to clinical cure (77.77%) (Table 1).

**Table 1:** Demographic and clinical characteristics of cutaneous leishmaniasis cases in the state of Paraná, Brazil, from 2001 to 2015 by the chi-squared test.

Characteristic	n	%	*p*-value
Gender			<0.001
Male	3,137	68.84	
Female	1,420	31.16	
Age Group (years)			<0.001
>1	33	0.72	
1–4	76	1.67	
5–9	184	4.04	
10–14	228	5.00	
15–19	281	6.17	
20–39	1,490	32.70	
40–59	1,435	31.49	
60–64	278	6.10	
65–69	228	5.00	
70–79	246	5.40	
≥80	78	1.71	
Clinical form			<0.001
Cutaneous	4,145	90.96	
Mucocutaneous	410	9.00	
Not informed	2	0.04	
Case outcome			<0.001
Clinical cure	3,544	77.77	
Abandonment of treatment	107	2.35	
Death related to CL	7	0.15	
Death of other cause	62	1.36	
Transfer	44	0.97	
Change diagnosis	44	0.97	
Not informed	749	16.44	

The occurrence of CL cases was verified in 268 municipalities (61.17%); of these, eight municipalities (2.99%) had a detection coefficient ≥ 71.0, 35 municipalities (13.06%) had a detection coefficient between 10.0 and 71.0, 95 municipalities (35.45%) had a detection coefficient between 2.5 and 10.0, and 130 municipalities (48.51%) had a detection coefficient <2.5.

Among the 268 municipalities in the state with registered cases of CL, the following were especially notable: 341 in Londrina (7.48%), 331 in Cianorte (7.26%), 279 in Cerro Azul (6.12%), 232 in Jussara (5.09%), 184 in Terra Boa (4.04%), 177 in Bandeirantes (3.88%), 158 in Adrianópolis (3.47%), 134 in Umuarama (2.84%), 111 in Japurá (2.44%), and 86 in Maringá (1.89%). Altogether, these municipalities comprised 44.61% of all cases of CL in the study period. The municipalities with the highest detection coefficients were Jussara (237.47), Adrianópolis (165.03), Cerro Azul (108.29), Ivatuba (108.11), and Japurá (89.35) (Table 2).

**Table 2:** Number and detection coefficient of cases of cutaneous leishmaniasis in municipalities with a detection coefficient >10.0 in the state of Paraná, Brazil, from 2001 to 2015.

Municipality	No. of cases	Detection coefficient*	Municipality	No. of cases	Detection coefficient*
Abatiá	22	19.13	Japurá	111	89.35
Adrianópolis	158	165.03	Jussara	232	237.47
Araruna	23	11.44	Lobato	10	15.29
Ariranha do Ivaí	9	23.58	Munhoz de Melo	6	11.21
Bandeirantes	177	35.85	Nova Tebas	11	10.58
Cambira	13	12.17	Pinhalão	25	26.49
Cândido de Abreu	54	20.88	Porto Rico	5	13.68
Carlópolis	82	39.69	Prudentópolis	81	11.21
Cerro Azul	279	108.29	Rio Bom	13	26.22
Cianorte	331	32.89	Rio Bonito do Iguaçu	75	32.67
Colorado	50	15.00	Sabaúdia	11	12.57
Conselheiro Mairinck	9	16.66	Santa Amélia	9	14.84
Corumbataí do Sul	12	19.53	São Carlos do Ivaí	12	12.65
Cruzeiro do Sul	8	11.56	São Jerônimo da Serra	67	39.26
Doutor Camargo	74	85.12	São Jorge do Ivaí	72	80.54
Doutor Ulysses	37	40.48	São Jorge do Patrocínio	39	45.26
Enéas Marques	12	13.24	São Tomé	49	61.74
Engenheiro Beltrão	71	33.81	Terra Boa	184	79.61
Grande Rios	14	13.01	Tomazina	47	35.25
Icaraíma	38	28.01	Tuneiras do Oeste	66	52.08
Itambaracá	32	32.68	Uniflor	7	19.47
Ivatuba	48	108.11	**Total**	2,685	38.69

*Cases per 100,000 inhabitants.

**Fig 2:**
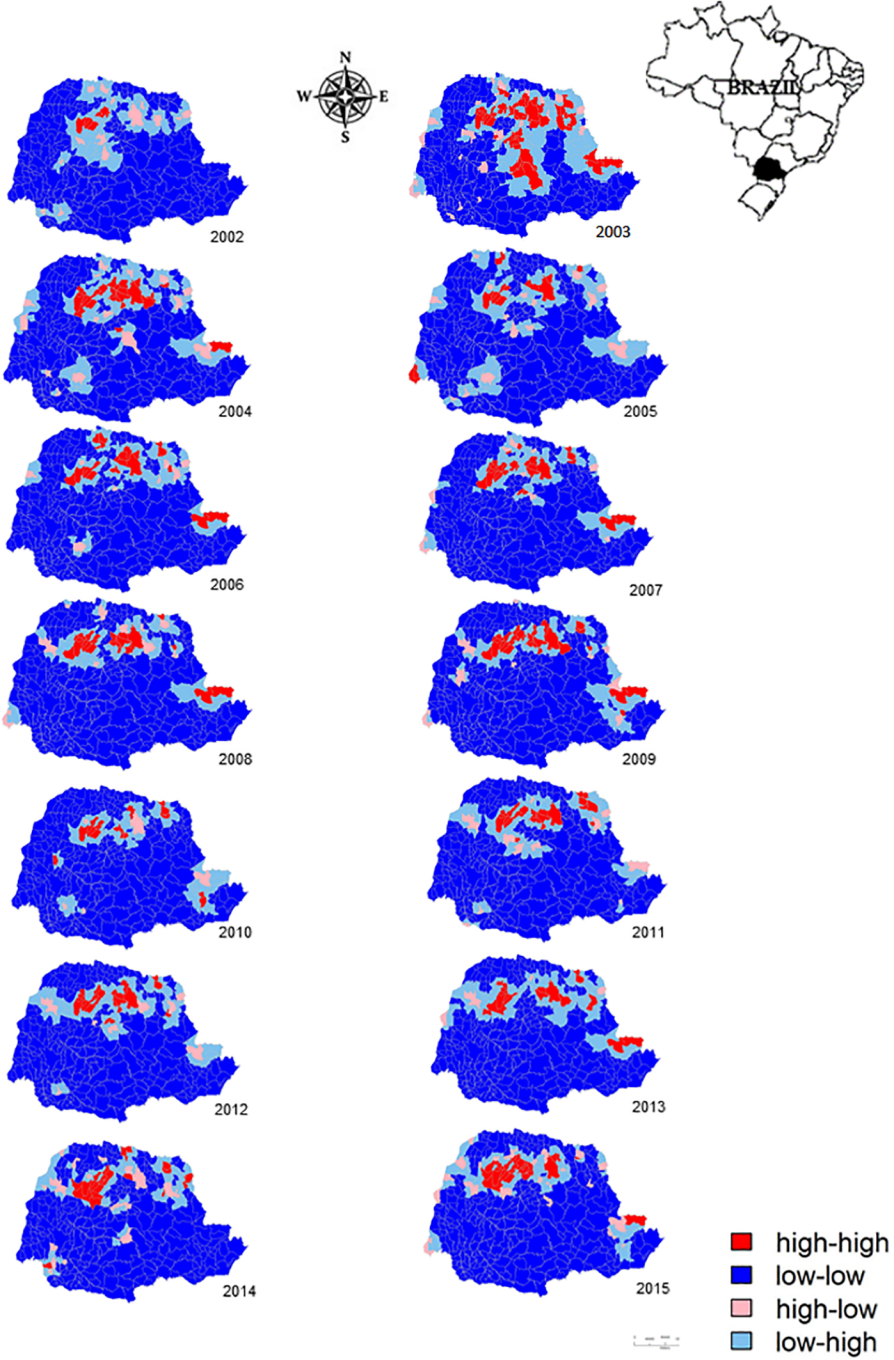
LISA cluster maps detailing the incidence of autochthonous cases of cutaneous leishmaniasis from 2002 to 2015. Darker areas indicate direct spatial autocorrelation. Lighter areas indicate negative autocorrelation.

The results of the global indices and their respective significance revealed no correlation in 2001 (I = -0.456, *p* = 0.676). In subsequent years, statistical evidence of a spatial correlation (Table 3) allowed the construction of LISA cluster maps (Fig 2). Fig 2 shows a high-high cluster area in municipalities in the Lower Ivaí Basin in each year from 2002 to 2015. High-high clusters were also observed in other basins, such as those of the Pirapó, Tibagi, and Ivaí Rivers. These were smaller in 2010 and 2014 and not evident in 2002. In the Ribeira River basin, a high-high association was found during the study years, except in 2002, 2005, 2010, 2011, 2012, and 2014. In 2010, Curitiba was a high-high cluster area, with a direct association for the occurrence of CL. In 2003, the highest number of municipalities with a direct association was found in the Ivaí River Basin and Pirapó River Basin. In the municipalities of the Lower Iguaçu River Basin, a positive association was found only in 2005 (Fig 2).

**Table 3:** Detection coefficient, Global Moran Index, and respective significance of cutaneous leishmaniasis in the state of Paraná, Brazil, between 2001 and 2015.

Year	Detection coefficient	Global Moran Index	*p*
2001	0.60	-0.456	0.676
2002	1.57	3.028	<0.01
2003	6.35	7.344	<0.01
2004	3.49	9.276	<0.01
2005	2.64	2.689	<0.01
2006	2.67	4.856	<0.01
2007	3.17	11.120	<0.01
2008	4.03	11.145	<0.01
2009	2.97	11.084	<0.01
2010	1.86	9.227	<0.01
2011	2.39	8.305	<0.01
2012	2.90	9.272	<0.01
2013	1.87	6.473	<0.01
2014	3.02	11.946	<0.01
2015	4.04	8.376	<0.01

## Discussion

The spatial distribution of CL in all Brazilian states shows the importance of this disease in the country [2]. The spatial distribution of CL demonstrates the significance of this disease throughout Brazil, with an increase in the number of cases in the 1980s and 1990s [2]. In Paraná, the disease has been registered in areas of ancient colonization, contrary to the expectation that the increase in human activities in the environment would result in the elimination of natural foci of CL [2].

The majority of infected individuals in Paraná during the study period were male and located in municipalities where the main economic activity is agriculture [6,7]. The most affected age group was 20–59 years, probably related to the agriculture work or recreational activities like fishing near riparian forests of rivers and streams where the enzootic cycle of *Leishmania* remains [5]. This was also observed in the municipality of Teodoro Sampaio in the state of São Paulo, Brazil [19], and in the country of Iran [20], which have features that are distinctive from Paraná, signifying that men may engage in behaviors that can lead to a higher risk of CL. Previous studies reported that the proportion of infected individuals is similar between agricultural and domestic workers [6,7]. The majority of urban residents acquired CL in Paraná during the study period in the rural area suggesting that pendulum migration is an important risk factor for CL in mesoregions north central, western center and northwest. In the state of Paraná [6]. The considerable number of women and children with CL that have been identified in studies in Paraná, including the present study, corroborate this assessment [6,7]. The number of female cases (31.15%), although less than males, is notable. Such cases appear to be more related to activities that are connected with agricultural work and the construction of residences and domestic animal shelters that are in close proximity to modified native forest where the environment is fresher and more pleasant [7].

Although cases of CL were registered in 268 municipalities, the municipalities with the highest detection coefficients were concentrated in the Ivaí-Pirapó CL hub, which is part of the Paraná-Paranapanema CL production circuit [7]. Examples of this are Jussara and Cianorte, which have large areas of moderately or highly altered residual forest and also secondary forests [5–7]. In the Alto Ribeira hub within the Ribeira circuit [7], the municipalities of Adrianópolis and Cerro Azul had elevated CL detection coefficients.

Londrina is part of the Ivaí-Pirapó hub. Although the municipality of Londrina had a detection coefficient <10 because of its larger population, it was responsible for the highest number of cases of CL between 2001 and 2015. A cluster of municipalities with high detection coefficients was identified in the Cinzas-Laranjinha area (Parana-Paranapanema circuit), one example of which is the municipality of Bandeirantes. Cases of CL were also registered in the municipalities of Cândido de Abreu and Prudentópolis in the Tibagi area (Paraná-Paranapanema circuit). A notable occurrence of cases was observed in the municipalities of the Lower Iguaçu area (Paraná-Paranapanema circuit), such as Rio Bonito do Iguaçu and Enéas Marques. Areas of high anthropogenic impact that is related to agriculture, especially corn, soybeans, sugar cane, and pasture, focus cases of CL in Paraná state [5].

The LISA map analysis revealed that only the municipalities of Jussara and Cianorte in the Ivaí-Pirapó area maintained a high-high association from 2002 to 2015. These municipalities play a major role in the production of CL in this area [7]. Previous studies have investigated *Leishmania* infection in dogs and wild animals in these areas [21–25]. The high number of phlebotomine sandflies in areas of altered native forest in the Ivaí-Pirapó area [3,26–28] may partially explain the persistence of the high-high cluster throughout the duration of the study period. Interestingly, the sandfly species *Nyssomyia neivai* (Pinto), *Nyssomyia whitmani* (Antunes & Coutinho), and *Migonemyia migonei* (França) were identified as *Leishmania* vectors [3,29,30], which are widely distributed in the state of Paraná [31]. In 2003, the high-high association covered all CL production areas in Paraná, with the exception of the Lower Iguaçu area.

The spatial analysis of the SINAN data from 2001 to 2015 allowed visualization of the local and global distribution, cluster formation, and spatial instability and identification of outliers of CL throughout the state of Paraná [32]. Beyond the use of georeferencing instruments, it is important to further investigate the migration of human populations and local conditions that influence the risk for CL in the referenced areas [33].

The public health surveillance should take into account the differences between the transmission patterns of each locality and the identified high-risk cluster in developing actions to mitigate the properties of the zoonosis in the state of Paraná. Moreover, the disease affects specific areas, such as Alto Ribeira, Ivaí-Pirapó, Tibagi, and Cinzas-Laranjinha. This suggests that health authorities need to provide information and develop campaigns regarding the importance of early diagnosis and treatment of CL, with the goal of reducing the emergence of new cases and preventing mucocutaneous cases of the disease. Moreover, the control of sandflies is essential to block the spread of the disease.

## Conclusion

The state of Paraná should keep a constant surveillance over cutaneous leishmaniasis due to the prominent presence of socioeconomic and environmental factors such as the favorable circumstances for the vectors present in peri-urban and agriculture areas.

Ivaí-Pirapó, Tibagi, Cinzas-Laranjinha, and Ribeira areas form the four-major hot spot CL areas in the state of Paraná.
